# Patient-Specific Alterations in CO_2_ Cerebrovascular Responsiveness in Acute and Sub-Acute Sports-Related Concussion

**DOI:** 10.3389/fneur.2018.00023

**Published:** 2018-01-24

**Authors:** W. Alan C. Mutch, Michael J. Ellis, Lawrence N. Ryner, Patrick J. McDonald, Marc P. Morissette, Philip Pries, Marco Essig, David J. Mikulis, James Duffin, Joseph A. Fisher

**Affiliations:** ^1^Department of Anesthesia and Perioperative Medicine, University of Manitoba, Winnipeg, MB, Canada; ^2^University of Manitoba, Winnipeg, MB, Canada; ^3^Canada North Concussion Network, University of Manitoba, Winnipeg, MB, Canada; ^4^Department of Surgery and Pediatrics and Child Health, University of Manitoba, Winnipeg, MB, Canada; ^5^Section of Neurosurgery, University of Manitoba, Winnipeg, MB, Canada; ^6^Pan Am Concussion Program, University of Manitoba, Winnipeg, MB, Canada; ^7^Childrens Hospital Research Institute of Manitoba, University of Manitoba, Winnipeg, MB, Canada; ^8^Department of Radiology Diagnostic Imaging, University of Manitoba, Winnipeg, MB, Canada; ^9^Division of Neurosurgery, BC Children’s Hospital, National Core for Neuroethics, University of British Columbia, Vancouver, BC, Canada; ^10^Pan Am Clinic Foundation, Winnipeg, MB, Canada; ^11^Max Rady College of Medicine, University of Manitoba, Winnipeg, MB, Canada; ^12^Department of Medical Imaging, University of Toronto, Toronto, ON, Canada; ^13^University of Toronto, Toronto, ON, Canada; ^14^University Health Network Cerebrovascular Reactivity Research Group, Toronto, ON, Canada; ^15^Department of Physiology, University of Toronto, Toronto, ON, Canada; ^16^Department of Anesthesia, University of Toronto, Toronto, ON, Canada

**Keywords:** sports-related concussion, adolescent, magnetic resonance imaging, blood oxygen level dependent imaging, cerebrovascular reactivity

## Abstract

**Background:**

Preliminary studies suggest that sports-related concussion (SRC) is associated with alterations in cerebral blood flow (CBF) regulation. Here, we use advanced magnetic resonance imaging (MRI) techniques to measure CBF and cerebrovascular responsiveness (CVR) in individual SRC patients and healthy control subjects.

**Methods:**

15 SRC patients (mean age = 16.3, range 14–20 years) and 27 healthy control subjects (mean age = 17.6, range 13–21 years) underwent anatomical MRI, pseudo-continuous arterial spin labeling (pCASL) MRI and model-based prospective end-tidal targeting (MPET) of CO_2_ during blood oxygenation level-dependent (BOLD) MRI. Group differences in global mean resting CBF were examined. Voxel-by-voxel group and individual differences in regional CVR were examined using statistical parametric mapping (SPM). Leave-one-out receiver operating characteristic curve analysis was used to evaluate the utility of brain MRI CO_2_ stress testing biomarkers to correctly discriminate between SRC patients and healthy control subjects.

**Results:**

All studies were tolerated with no complications. Traumatic structural findings were identified in one SRC patient. No significant group differences in global mean resting CBF were observed. There were no significant differences in the CO_2_ stimulus and O_2_ targeting during BOLD MRI. Significant group and patient-specific differences in CVR were observed with SRC patients demonstrating a predominant pattern of increased CVR. Leave-one-out ROC analysis for voxels demonstrating a significant increase in CVR was found to reliably discriminate between SRC patients and healthy control subjects (AUC of 0.879, *p* = 0.0001). The optimal cutoff for increased CVR declarative for SRC was 1,899 voxels resulting in a sensitivity of 0.867 and a specificity of 0.778 for this specific ROC analysis. There was no correlation between abnormal voxel counts and Postconcussion Symptom Scale scores among SRC patients.

**Conclusion:**

Acute and subacute SRCs are associated with alterations in CVR that can be reliably detected by brain MRI CO_2_ stress testing in individual patients.

## Introduction

Expert consensus has historically viewed sports-related concussion (SRC) as an injury resulting in clinical features that arise from transient disturbances in brain functioning occurring in the absence of structural brain injury (1). Clinical studies indicate that the majority of adolescents and adults who sustain a SRC achieve symptomatic and neurocognitive recovery within 1–4 weeks postinjury (1–3). However, approximately 10–30% of SRC patients will develop persistent postconcussion symptoms (4, 5) that are mediated by heterogeneous pathophysiological processes that remain poorly understood (6). While advanced neuroimaging studies have provided some insight into these pathophysiological processes, results to date have been largely limited to the detection of differences between groups of SRC patients and healthy control subjects (7, 8). As such, there remains an urgent unmet need for novel neuroimaging tools that can provide qualitative and quantitative assessment of these processes in individual SRC patients.

Among the processes implicated in the pathogenesis of acute concussion and traumatic brain injury (TBI) are disturbances in cerebrovascular physiology that result in a mismatch between the metabolic demands of the injured brain and the delivery of cerebral blood flow (CBF) (9–13). One of the means to assess the physiological capacity of the cerebral vasculature to respond appropriately during periods of metabolic stress or injury is cerebrovascular reactivity or responsiveness (CVR). CVR is defined as the change in CBF that occurs in response to a vasoactive stimulus (14, 15). Accurate measurement of CVR can be achieved by the administration of a precise, quantifiable, and reproducible vasodilatory stimulus while measuring CBF or its surrogate (16, 17). Using model-based prospective end-tidal targeting (MPET) of CO_2_ during blood oxygenation level-dependent (BOLD) magnetic resonance imaging (MRI), we have previously demonstrated patient-specific qualitative and quantitative regional alterations in CVR among adolescent postconcussion syndrome patients while global mean resting CBF remained within normal limits (18). Although these results suggest that the cerebrovascular disturbances that characterize acute SRC can persist into the chronic phases of injury, available studies that have aimed to evaluate alterations in CVR using various techniques among acute and subacute SRC patients have yielded inconsistent results (19–21).

In this study, we examined group differences in global mean resting CBF and CVR in healthy control subjects and adolescent acute and subacute SRC patients. Using a leave-one-out receiver operating characteristic (ROC) analysis we examined individual differences in CVR among SRC patients compared to an institutional atlas of healthy adolescent control subjects assessed using the same neuroimaging assessment technique.

## Materials and Methods

### Research Design and Clinical Assessment

We conducted a case–control study of adolescent SRC patients that were compared to an institutional atlas of healthy control subjects that includes subjects used in previous studies conducted by our group (18, 22). Adolescent SRC patients were recruited from the Pan Am Concussion Program, a multi-disciplinary pediatric concussion clinic that accepts referrals for children and adolescents with sports- and non-sports-related TBI in Winnipeg, MB, Canada. All adolescent SRC patients included in the study underwent a clinical assessment by a single neurosurgeon. Patient inclusion criteria for this study included: (1) physician diagnosis of SRC according to the definition of the International Consensus on Concussion in Sport guidelines (1); (2) age 13–21 years; and (3) patients had to be symptomatic at rest or during exercise at the time of neuroimaging assessment. All healthy control subjects underwent a clinical interview to collect demographic, past medical history, and past concussion history data. Healthy control subject inclusion criteria for the study included: (1) age 13–21 years. Exclusion criteria for control subjects included: (1) the presence of a symptomatic concussion; (2) diagnosis of prior moderate or severe TBI or neurological condition resulting in structural brain abnormality detected on prior neuroimaging; (3) contra-indication to MRI (i.e., dental braces, claustrophobia); and (4) diagnosis of a neurological condition requiring prescription medication.

In general, SRC patients were deemed clinically recovered when they were asymptomatic at rest, were back to full-time school, had a normal neurological examination, and successfully completed the International Consensus on Concussion in Sport return-to-play guidelines (1). Some patients underwent graded aerobic treadmill testing as part of their clinical management to help classify patients into post-traumatic clinical subtypes and inform the design of multidisciplinary rehabilitation strategies. Patients were diagnosed with autonomic/physiological postconcussion disorder (PCD) if they demonstrated a symptom-limited threshold on graded aerobic treadmill testing as previously described (6, 23).

Institutional research ethics approval at the University of Manitoba was obtained for this study. Informed patient, and where applicable, parental consent was obtained for all participants prior to participating in the study. On the day of neuroimaging, all subjects completed the Postconcussion Symptom Scale (PCSS), a concussion symptom inventory consisting of 22 symptoms that are rated on a 7-point (0–6) Likert scale with a maximum score of (6 × 22) = 132 (24).

### MRI Assessment

Prior to neuroimaging assessment, all study subjects were exposed to a short trial of the breathing sequence to familiarize them with the changes in breathing that would occur while in the MRI scanner. During MRI assessment, subjects underwent non-invasive heart rate, pulse oximetry, and blood pressure monitoring. Subjects were also given a hand-held call button that allowed them to voluntarily terminate the test at any time. Neuroimaging assessment in this study consisted of (1) anatomical imaging; (2) BOLD CVR mapping; and (3) global resting CBF measurements that were all obtained in same order.

#### Imaging Acquisition

Images were acquired using a Siemens Verio 3.0 T MR scanner with a 12-channel phased-array head coil. Anatomical imaging was acquired without manipulation of end-tidal gases using a sagittal 3D T1 MPRAGE (whole brain coverage; matrix: 256 × 256; slice thickness: 2.2 mm; no interslice gap; voxel size 2 mm × 2 mm × 2 mm) and axial gradient recalled echo planar (GRE) sequences to screen for cerebral microhemorrhages as well as GRE B0-field mapping. CVR was assessed using continuous BOLD MRI during MPET CO_2_ targeting. BOLD MRI data were acquired using a T2*-weighted single-shot gradient echo pulse sequence with echoplanar readout (field of view: 24 cm × 24 cm; matrix: 64 × 64; TR: 2,000 ms; TE: 30 ms; flip angle: 85°; slice thickness: 5.0 mm; interslice gap: 2.0 mm; voxel size 3.75 × 3.75 × 6 mm; number of temporal frames = 330; 10 s of initial imaging data was discarded to allow for equilibration). Global mean resting CBF was assessed using pseudo-continuous arterial spin labeling (pCASL) that included an initial M0 scan—Siemens ep2d_pCASL—echo planar readout (field of view 24 cm × 24 cm, TR 8,000 ms, TE 12 ms, contrast with a flip angle 90°, 20 slices, CASL method—multislice, label offset 90 mm, post label delay 1,200 ms, crusher gradient 0 s/mm^2^, and voxel size 3.8 mm × 3.8 mm × 5.0 mm). The formal pCASL sequence then followed consisting of an echo planar readout (field of view 24 cm × 24 cm, TR 4,000 ms, TE 12 ms, contrast with a flip angle 90°, 20 slices, slice thickness 5.0 mm, CASL method—multislice, label offset 90 mm, post label delay 1,200 ms, crusher gradient 0 s/mm^2^; voxel size 3.8 mm × 3.8 mm × 5.0 mm). Imaging duration was for 3 min. The first two labeled–non-labeled pairs were discarded.

#### Vasodilatory Stimulus

**Figure 1:**
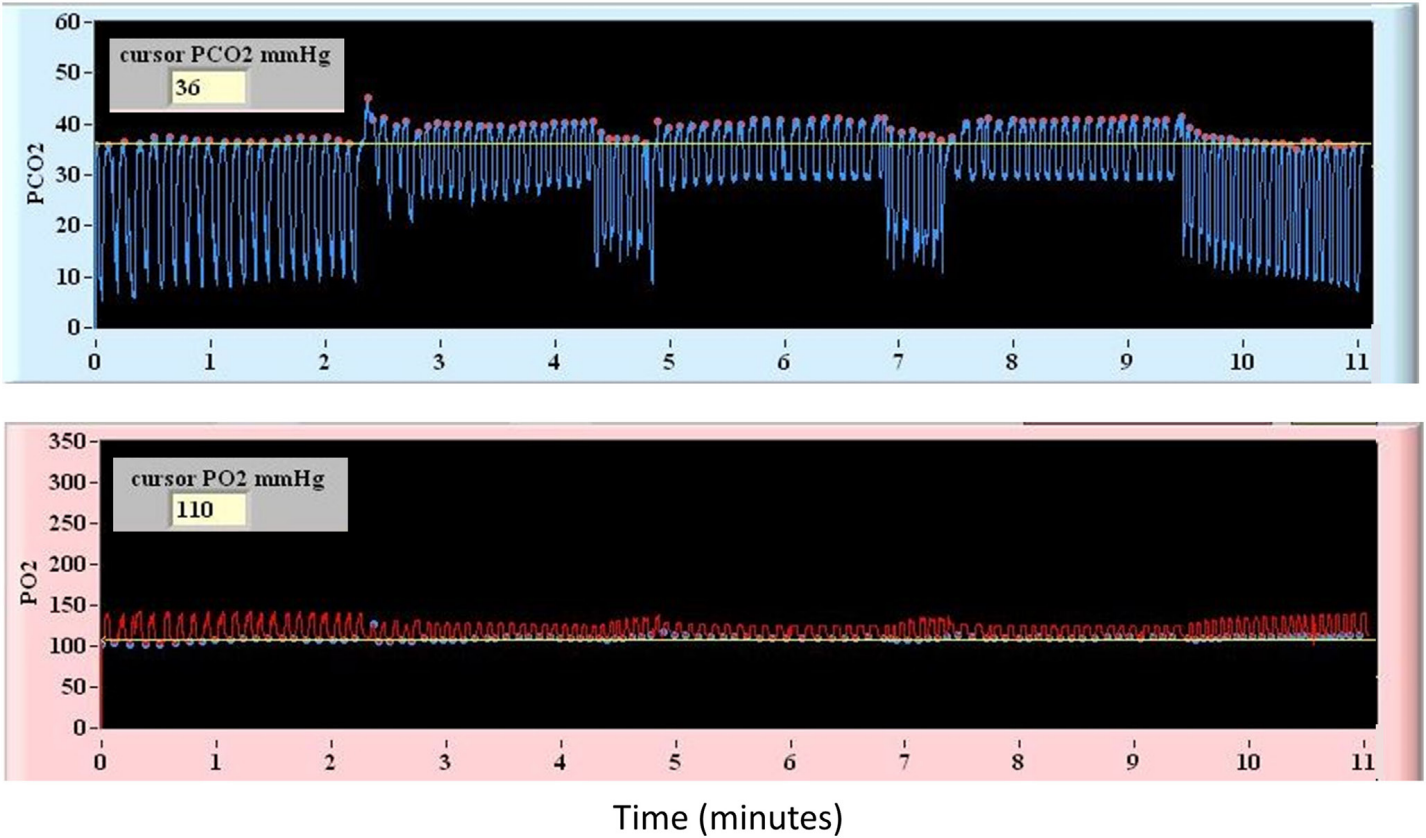
Representative diagram of the controlled square-wave triple hypercapnic stimulus under isoxic conditions used during blood oxygenation level-dependent magnetic resonance imaging.

The vasodilatory stimulus used for CVR mapping in this study consisted of precise delivery of CO_2_ and O_2_ *via* a computer-controlled gas blender (RespirAct, Thornhill Research Inc., Toronto, ON, Canada) connected to a rebreathing circuit and mask secured to the subject’s face. This device allows MPET targeting of P_ET_CO_2_ under isoxic conditions while providing continuous breath-by-breath measurement of P_ET_CO_2_ and P_ET_O_2_. Previous work has demonstrated that measurement of P_ET_CO_2_ is an accurate surrogate for PaCO_2_ during MPET CO_2_ targeting (25). The breathing sequence used for CVR mapping in all patients included a triple stimulus square-wave sequence consisting of interval step changes as follows: baseline P_ET_CO_2_ (120 s), hypercapnia (5 mmHg above baseline for 120 s), baseline P_ET_CO_2_ (30 s), hypercapnia (5 mmHg above baseline for 120 s), baseline P_ET_CO_2_ (30 s), hypercapnia (5 mmHg above baseline for 120 s), and baseline P_ET_CO_2_ (120 s). During this sequence a stable P_ET_O2 of 115 mmHg was targeted. A representative illustration of the breathing sequence is provided in Figure 1.

### Data Processing

Prior to data processing, subject head motion artifact and end-tidal CO_2_ targeting were examined. Study subjects were excluded if motion over the course of the imaging acquisition was greater than 3 mm in any plane or if inadequate end-tidal CO_2_ targeting was identified during BOLD MRI acquisition.

#### Preprocessing of MRI Sequences

Standard preprocessing of MRI EPI output was carried out for the BOLD MRI data using SPM8 software including batch processing by a statistical parametric mapping (SPM) toolbox and custom written in-house MatLab scripts. The BOLD data were interpolated to the MPRAGE voxel dimensions. The fMRI model specification was a two-pass process. First, the data were modeled using the finite impulse response package in SPM. The MPET triple stimulus square-wave sequence was assessed with a zero offset. An event-related response was calculated by examining the whole brain response to the CO_2_ stimulus. The time to maximal response to the CO_2_ stimulus was corrected based on a constructed series of time delay block stimulus files generated with delays from 0 to 30 s. Second, following determination of the time delay for the CO_2_ stimulus to the brain the newly generated time delay-based fMRI model was rerun as above.

Further preprocessing was conducted using a motion correction file generated with realignment as regressors in the model. First-level CVR analyses were undertaken using the time-corrected analysis, and the contrast images generated were then used in the second level CVR analysis. In order to account for BOLD EPI signal inhomogeneities at the skull base and within the choroid plexus and periventricular white matter (26), individualized masks were constructed as previously described (22). Masked images [labeled (gm + wm) − (B0 + dil_ventricles)] were used in the second-level CVR analysis to ensure that the abnormal voxels for each individual study were intraparenchymal and not contaminated by these other sources of BOLD EPI signal inhomogeneities. First-level CVR analyses were undertaken with the author blinded to the subject’s group (healthy control subject versus SRC patient); however, second-level CVR analyses were not blinded since they were based on the results of the first-level analysis.

An ASL toolbox was used to undertake preprocessing of the pCASL data (http://www.cfn.upenn.edu/~zewang/ASLtbx.php).

### Statistical Analysis

#### Anatomical Imaging

Anatomical imaging was reviewed and reported by a licensed neuroradiologist.

#### Global Mean Resting CBF

Cerebral blood flow was calculated on a voxel-by-voxel basis at rest and used to calculate a global mean resting CBF for all study subjects.

#### Cerebrovascular Responsiveness (First-Level Analysis)

First-level analysis for the unmasked CVR data was undertaken for each study participant. BOLD increases and decreases in response to the MPET triple stimulus square-wave sequence was assessed at the *p* = 0.001 level. The cluster size threshold was 10 voxels.

#### Cerebrovascular Responsiveness (Second-Level Analysis)

Second-level analysis for the CVR data was completed among individual SRC patients and control subjects. Voxel-by-voxel comparisons on a group and individual basis for the SRC patients was conducted at the *p* = 0.005 level to identify voxels that responded less than or greater than the mean healthy control group responses to the CO_2_ stimulus from the atlas (i.e., abnormal voxel counts). For each individual second-level comparison, the images were masked using the combined individual mask described above versus the atlas output. Voxel counts calculated for each subject were based on the applied individual mask. For the control subjects each of their contrast images were removed in turn (leave one out) from the atlas to determine the second-level voxel counts for that control subject. We report on the *p* = 0.005 values for each patient and subject.

#### Leave-One-Out ROC Curve Analysis

Leave-one-out ROC analyses were undertaken to determine whether abnormal voxel counts derived from the second level CVR analysis could accurately discriminate between individual SRC patients and the leave-one-out results determined for each healthy control subject on a quantitative basis. Second-level analysis voxel counts for each individual (leave one out for that individual versus the rest of the control atlas subjects and each individual SRC patient versus the complete control atlas) were determined based on the *p* = 0.005 greater than and less than voxel counts for each individual. The ROC curves were constructed comparing the leave one out counts for each control subject (SRC = 0) and the counts calculated for each SRC patient (SRC = 1). The Analyse-It statistical package for Excel 2010 was used to calculate the ROC AUC values for these voxel counts.

## Results

### Participants

A total of 55 subjects were enrolled in the study including 37 healthy control subjects and 18 SRC patients; however, subject data for 7 controls and 3 SRC patients were excluded due to excessive motion, and data for 3 controls were excluded due to poor end-tidal CO_2_ targeting. Therefore, 27 healthy control subjects and 15 SRC patients were included in the final analysis. Two acute SRC patients included in a prior study of longitudinal CVR imaging were included (22). Baseline characteristics of adolescent healthy control subjects and SRC patients are summarized in Table 1. Additional clinical data on the individual SRC patients is summarized in Table 2.

**Table 1:** Baseline characteristics of adolescent healthy control subjects and sports-related concussion patients.

	Healthy control subjects (*N* = 27)	Sports-related concussion patients (*N* = 15)
Mean age in years (range)	17.6 (13–21)	16.3 (14–20)
Male	13 (48%)	9 (60%)
Past medical history of previous concussion	5 (19%)	12 (80%)
Past medical history of migraine	1 (4%)	2 (13%)
Past medical history of depression	0 (0%)	1 (7%)
Mean PCSS score at time of neuroimaging assessment (range)	1 (0–7)	42 (1–82)
Mean days from injury to neuroimaging assessment (range)	N/A	16 (3–32)
Sport played at the time of injury	N/A	
Hockey		6 (40%)
Football		4 (27%)
Basketball		1 (7%)
Rugby		1 (7%)
Volleyball		1 (7%)
Soccer		1 (7%)
Ringette		1 (7%)

*PCSS, Postconcussion Symptom Scale; N/A, not applicable*.

**Table 2:** Summary of demographic, clinical features, outcomes, and quantitative brain magnetic resonance imaging CO_2_ stress testing results in sports-related concussion patients.

Subject	Past medical history	Time from injury to imaging assessment (days)	PCSS score at time of imaging assessment	Abnormal voxel counts (at imaging assessment, *p* < 0.005)	Clinical outcomes
1	3 previous concussions	29	8	Greater: 19,973 Less: 370 Total: 20,343	– Autonomic/physiological PCD – Required medication for post-traumatic headaches – Remained symptomatic at last follow-up (154 days)

2	1 previous concussion	13	82	Greater: 21,008 Less: 268 Total: 21,276	– Clinically recovered (41 days postinjury)

3	2 previous concussions	3	1	Greater: 12,885 Less: 176 Total: 13,061	– Clinically recovered (9 days postinjury)

4	1 previous concussion	27	58	Greater: 1,002 Less: 0 Total: 1,002	– Autonomic/physiological PCD – Remained symptomatic at last follow-up (90 days postinjury)

5	1 previous concussion	7	69	Greater: 2,091 Less: 210 Total: 2,301	– Clinically recovered (34 days postinjury)

6	2 previous concussions, migraine headaches	6	46	Greater: 1,607 Less: 49 Total: 1,656	– Autonomic/physiological PCD – Remained symptomatic at last follow-up (40 days postinjury)

7	2 previous concussions	7	18	Greater: 35,429 Less: 93 Total: 35,522	– Autonomic/physiological PCD – Clinically recovered (140 days postinjury)

8	None	22	56	Greater: 14,695 Less: 11 Total: 14,706	– Autonomic/physiological PCD – Clinically recovered (211 days postinjury)

9	3 previous concussions, depression	4	24	Greater: 12,575 Less: 16 Total: 12,591	– Remains in treatment – Abnormal formal neuropsychological testing (196 days postinjury)

10	2 previous concussions	12	56	Greater: 16,846 Less: 45 Total: 16,891	– Autonomic/physiological PCD – Remained symptomatic at last follow-up (60 days postinjury)

11	1 previous concussion	8	48	Greater: 7,772 Less: 66 Total: 7,838	– Clinically recovered (21 days postinjury)

12	None	27	23	Greater: 4,199 Less: 20 Total: 4,219	– Autonomic/physiological PTD – Clinically recovered (84 days postinjury)

13	None	25	42	Greater: 5,717 Less: 694 Total: 6,411	– Autonomic/physiological PCD – Clinically recovered (39 days postinjury)

14	1 previous concussion	32	20	Greater: 9,890 Less: 0 Total: 9,890	– Autonomic/physiological PCD – Clinically recovered (101 days postinjury)

15	3 previous concussions, migraine headaches	18	71	Greater: 20,331 Less: 15 Total: 20,346	– Autonomic/physiological PCD – BPPV – Clinically recovered (55 days postinjury)

*PCSS, Postconcussion Symptom Scale; M, male; F, female; PCD, postconcussion disorder; BPPV, benign paroxysmal positional vertigo*.

#### Study Tolerability

All neuroimaging assessments were completed without any serious adverse events. No study subjects voluntarily terminated the test. Three SRC patients developed transient and self-limiting symptoms during neuroimaging assessment including mild headache, lightheadedness, and fatigue. None of these patients required any medical attention for these symptoms.

#### Anatomical Imaging

Anatomical imaging studies were normal in 26/27 (96%) of healthy control subjects with one patient found to have a right frontal developmental venous anomaly. Anatomical imaging studies were normal in 11/15 (73%) of SRC patients. Abnormalities found among SRC patients included a left frontal developmental venous anomaly (one patient), pineal cyst (one patient), non-functioning pituitary microadenoma (one patient), and a traumatic microhemorrhage located in the splenium of the corpus callosum that was identified on imaging obtained following a previous concussion (one patient, Subject 3 in Table 2).

#### End-Tidal Gas Targeting, Global Mean Resting CBF, and Cerebrovascular Responsiveness (Second-Level Analysis)

Group comparisons of end-tidal gas targeting and global mean resting CBF are summarized in Table 3. Overall, there were no differences in mean baseline P_ET_CO_2_, mean change in P_ET_CO_2_ during MPET CO_2_ targeting, and mean P_ET_O_2_ over the period of the CO_2_ challenge between the SRC patients and healthy control subjects. These results indicate that both groups were exposed to the same magnitude of hypercapnic stimulus under isoxic conditions during neuroimaging assessment.

**Table 3:** Group comparisons of end-tidal gas targeting and global mean resting cerebral blood flow.

	Healthy control subjects (*N* = 27)	Sports-related concussion patients (*N* = 15)	*p*-Value
Mean baseline P_ET_CO_2_ ± SD (mmHg)	40.6 ± 4.1	40.7 ± 4.4	0.95
Mean change in P_ET_CO_2_ during MPET CO_2_ targeting ± SD (mmHg)	4.0 ± 0.9	4.0 ± 1.1	0.98
Mean P_ET_O_2_ over the period of the CO_2_ challenge ± SD (mmHg)	112.6 ± 3.2	112.6 ± 2.4	0.94
Global mean resting cerebral blood flow ± SD (mL/100 g/min)	41.0 ± 9.7	36.4 ± 7.0	0.13

*P_ET_CO_2_, end-tidal carbon dioxide; P_ET_O_2_, end-tidal oxygen; MPET, model-based prospective end-tidal targeting*.

There were no significant group differences in global mean resting CBF among SRC patients and healthy control subjects.

**Figure 2:**
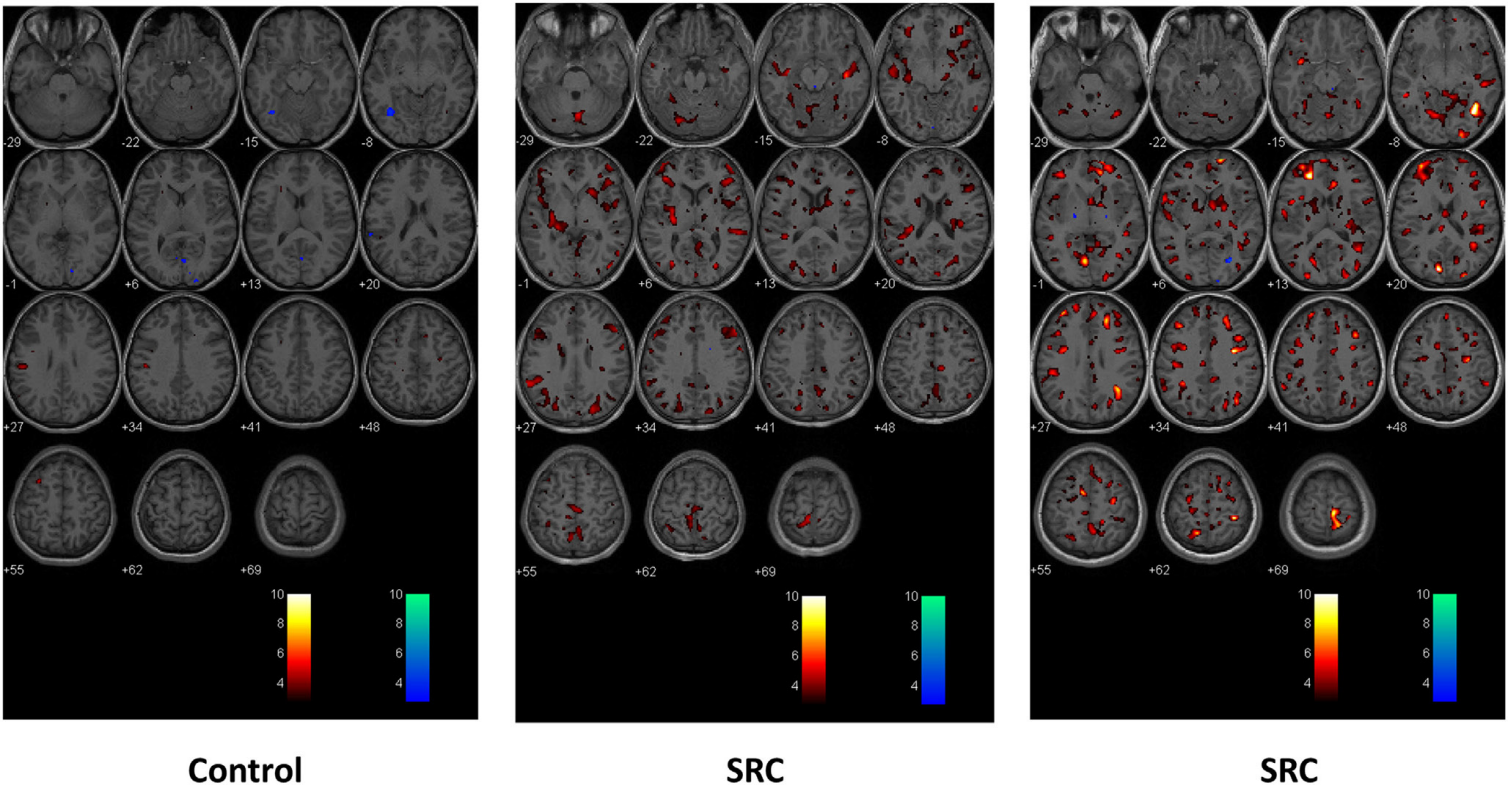
Second-level comparisons of one individual control subject (left panel) and two sports-related concussion (SRC) patients (middle and right panel) against the normal control atlas. Examined at the *p* = 0.005 level. Global and regional significant differences in voxel-by-voxel responses that were greater than and less than the mean healthy control atlas response are indicated in color. Hot scale *t*-statistic level (orange hues) indicates where the individual subject had a greater response to the CO_2_ stimulus when compared to the control group; cold scale *t*-statistic level (blue hues) indicates where the individual subject had a diminished response to the CO_2_ stimulus compared to the control group.

**Figure 3:**
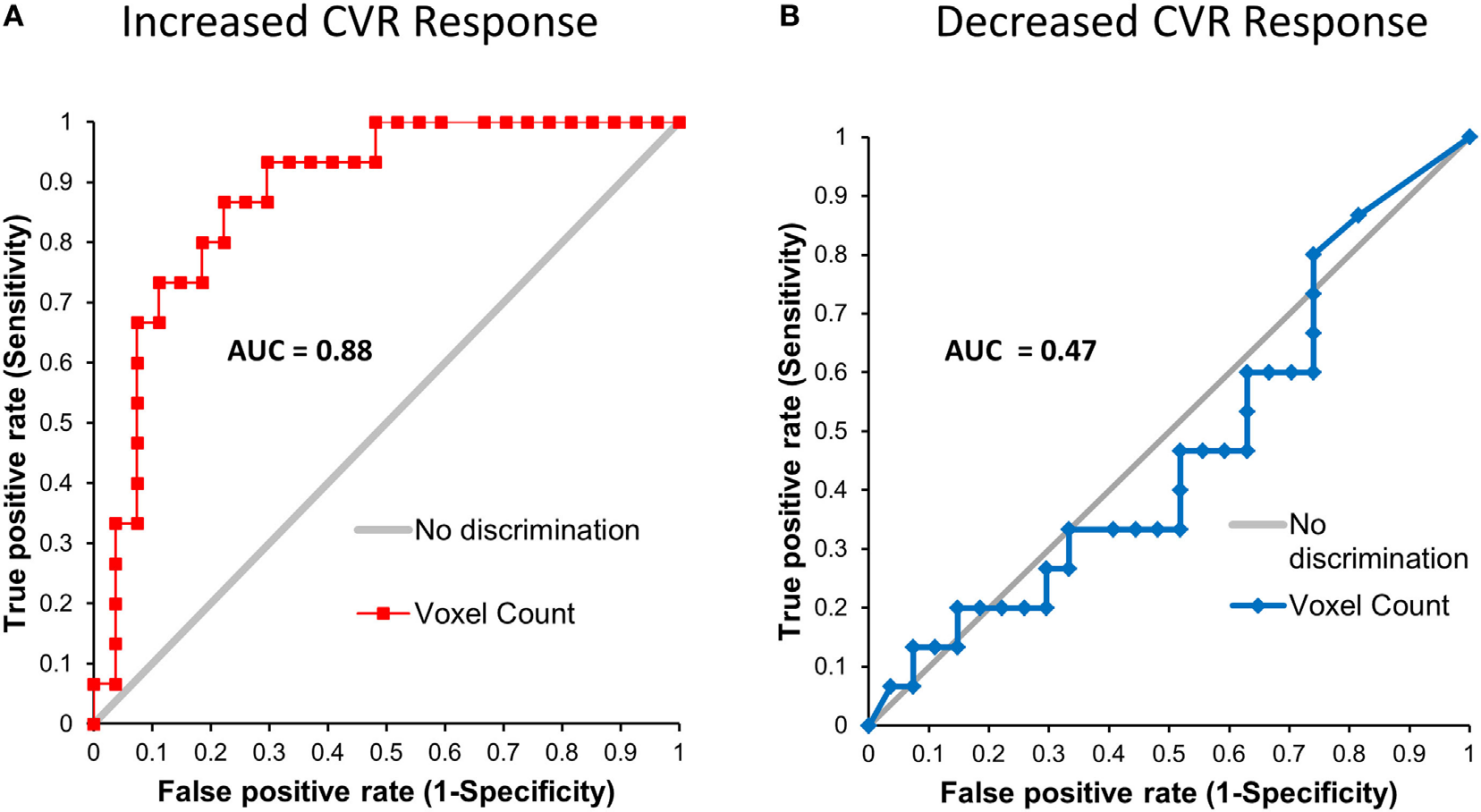
Leave-one-out receiver operating characteristic curves for voxels manifesting a blood oxygenation level-dependent magnetic resonance imaging response to the CO_2_ stimulus that was greater than **(A)** and less than **(B)** the mean response of the control atlas at the *p* = 0.005 level.

First- and second-level group comparisons of unmasked CVR data are summarized in Table 4. At the *p* = 0.001 level, there were no significant group differences in activation between the SRC patients and healthy controls suggesting that a consistent vasoactive stimulus was applied to each group. Second-level group comparisons between the SRC patients and normal control subjects at the *p* = 0.005 level revealed significant differences in CVR with SRC patients demonstrating a predominant pattern of increased CVR resulting in significantly higher total abnormal voxel counts compared to healthy controls. The absolute CVR (deltaBOLD/deltaCO_2_) was 0.223 ± 0.127 for the SRC patients and 0.139 ± 0.079 for the control subjects (see Figures S1 and S2 in Supplementary Material for additional details). Second-level analysis comparing individual SRC patients to the healthy control atlas demonstrated patient-specific alterations in CVR (Figure 2). The ROC curve for brain regions manifesting a response less than (AUC = 0.468; *p* = NS) and greater than (AUC = 0.879; p < 0.0001) mean control group responses at the second-level analysis voxel counts at the *p* = 0.005 level is shown in Figure 3. The optimal cutoff for increased CVR declarative for SRC was 1,899 voxels resulting in a sensitivity of 0.867 and a specificity of 0.778 for this specific ROC analysis. There was no correlation between abnormal voxel counts and PCSS scores among SRC patients (see Figures S1 and S2 in Supplementary Material).

**Table 4:** First- and second-level group comparisons of CO_2_ cerebrovascular responsiveness.

First-level analysis (reported at *p* = 0.001)					

Group (mean ± SD)	Hyper response	Inverse response	Absolute voxel count	% hyper response	% inverse response
Controls	149,305 ± 11,399	1,285 ± 1,012	186,452 ± 5,179	80.1 ± 6.3	0.7 ± 0.6
Sports-related concussion patients	145,055 ± 25,767	1,119 ± 1,106	186,033 ± 5,168	77.9 ± 13.0	0.6 ± 0.6
*t*-Test	0.46	0.63	0.80	0.45	0.64

**Second level analysis (reported at *p* = 0.005)**					

**Group (mean ± SD)**	**Increased CVR response**	**Decreased CVR response**	**Abnormal voxel count**	**% CVR increase**	**% CVR decrease**

Controls	2,964 ± 5,820	221 ± 248	3,185 ± 5,881	1.6 ± 3.1	0.12 ± 0.13
Sports-related concussion patients	13,413 ± 10,092	202 ± 286	13,615 ± 10,129	7.2 ± 5.4	0.11 ± 0.15
*t*-Test	0.001	0.82	0.002	0.001	0.82

## Discussion

This study examined global mean resting CBF and global and regional CVR in adolescent SRC patients and healthy controls subjects. In a heterogeneous population of SRC patients who underwent neuroimaging assessment at various stages of acute and subacute injury, we observed significant group differences in CVR that occurred in the setting of normal global mean resting CBF. When compared to an atlas of healthy control subjects, we found that SRC patients demonstrated significant patient-specific alterations in CVR with a predominant pattern of increased CVR including those patients who were found to demonstrate exercise intolerance during graded aerobic treadmill testing and were diagnosed with autonomic/physiological PCD. Furthermore, a preliminary leave-one-out ROC analysis utilizing the quantitative biomarker of increased CVR response (voxels demonstrating an increased response to a controlled hypercapnic stimulus) generated from brain MRI CO_2_ stress testing was found to reliably discriminate patients with and without SRC with an ROC AUC of 0.879 (*p* = 0.0001). At a cut-point of approximately 1,900 voxels for increased CVR derived from our second-level analysis at the 0.005 level, this biomarker resulted in a sensitivity of 87% and a specificity of 78% for correctly identifying patients with SRC. There was no discrimination seen for a decreased CVR response for these SRC patients (AUC = 0.468). Importantly, all neuroimaging studies were well tolerated with no complications.

At present, the natural history of CVR impairments following SRC remains poorly understood due in large part to the relative paucity of studies that have examined CVR during the acute, subacute, and chronic stages of injury. Among available studies, Len et al. (19) examined CVR in 21 healthy controls and 10 SRC patients within 7 days of injury using transcranial Doppler ultrasonography of the right middle cerebral artery (MCA) and a breath-holding and hyperventilation challenge. No significant differences in percentage change in MCA velocity were observed between the SRC and control groups during breath-holding; however, greater reductions in percent-grouped mean MCA velocity were observed among SRC patients during the hyperventilation challenge. Using the same technique, Len et al. (20) examined CVR in 20 SRC patients at 2, 4 and 8 days postinjury including 18 that underwent baseline preseason or postseason evaluations. During breath-holding mean MCA velocity was found to be increased on day 2 and decreased on days 4 and 8 with less robust findings observed during hyperventilation. Militana et al. (21) examined CVR in 11 healthy controls and six SRC patients 3–6 days postinjury by measuring alterations in BOLD MRI signal during an inhaled CO_2_ challenge. In the SRC group, they observed increased CVR for all preselected regions of interests including significant increases within the median default mode network and the anterior cingulate. They also found that increased CVR in the hippocampus was positively correlated with increased functional connectivity between the hippocampus and precuneus in the SRC group but not in the healthy control group. The authors also found no group differences in resting mean CBF between groups similar to the results of our present study. Finally, in a previous study our group examined CVR in 17 healthy control subjects and 15 adolescent SRC patients who remained symptomatic during the chronic phase of injury (33–993 days postinjury) (18). Using MPET of CO_2_ and BOLD MRI we observed significant group differences in CVR characterized by a predominant pattern of decreased CVR when group means were compared. More importantly when compared to the healthy controls on an individual basis, we found that each SRC patient demonstrated a unique signature of both increased and decreased regional CVR even in the setting of normal global mean resting CBF.

The differences in CVR findings observed across the available studies are undoubtedly impacted by the techniques used to measure CVR. To achieve accurate within- and between-subject measures of CVR requires a precise, quantifiable, and reproducible vasodilatory stimulus to be administered to all study participants during assessment of CBF. Although transcranial Doppler and BOLD MRI are commonly used techniques to assess cerebral blood velocity and CBF, respectively, the vasoactive stimulus chosen to provide the stress for the CVR study each have their own strengths and limitations. The most common techniques used to provide a vasoactive stimulus in CVR studies include breath-holding, inhaled CO_2_, and MPET CO_2_ targeting (13, 16). Breath-holding is one of the most widely used methods to induce hypercapnia due to ease of use and lack of requirement for specialized personnel or equipment. Unfortunately, breath-holding fails to result in a linear rise in the arterial partial pressure of carbon dioxide (PaCO_2_) over time (27); the P_ET_CO_2_ detected at the end of breath-holding (the measured stimulus) is not always equal to the PaCO_2_ (the stimulus acting on the cerebral vasculature); and P_ET_CO_2_ and P_ET_O_2_ levels cannot be sampled throughout the breath-holding periods (16). Together, these limitations can lead to significant differences in the vasodilatory stimuli administered across groups and subjects thereby impacting CVR measurement and contributing to experimental noise. This finding is illustrated in the study by Len et al. (19) discussed above, where mean P_ET_CO_2_ levels of 41.2 and 37.9 mm Hg among healthy controls and SRC patients, respectively, were obtained during breath-holding. An alternative method for providing a hypercapnic stimulus is inhaled CO_2_. Although this technique relies on the administration of a gas containing a fixed fractional concentration of CO_2_, the resultant effect on PaCO_2_ has also been found to vary considerably between subjects and within the same subject over serial administrations (28, 29). Importantly, both breath-holding and inhaled CO_2_ techniques can also lead to changes in PaO_2_ that can result in independent and unaccounted for changes in CBF and CVR, especially with BOLD MRI approaches. In the study by Militana et al. (21) utilizing inhaled CO_2_, resting P_ET_CO_2_ levels at baseline and during the CO_2_ challenge were not reported. In contrast, the MPET CO_2_ used here and in our previous studies (18, 22) has emerged as the only technique that is capable of providing a precise, quantifiable, and reproducible CO_2_ stimulus under controlled isoxic conditions (16). This technique employs end-inspiratory rebreathing that ensures that the P_ET_CO_2_ is equivalent to the PaCO_2_ (25) as well as continuous breath-by-breath sampling (30). By combining this vasodilatory stimulus with BOLD MRI and standardized data analysis techniques, brain MRI CO_2_ stress testing results in highly reliable within (15, 31) and between subject measures of CVR that can be quantified on an individual subject basis (13, 17, 32). In this study, we observed no significant group differences in baseline P_ET_CO_2_ or P_ET_CO_2_ and P_ET_O_2_ during MPET targeting suggesting the results of brain MRI CO_2_ stress testing obtained here are indeed a true reflection of abnormal CVR in these patients and are not due to variations in the vasoactive stimuli applied to these subjects.

Taken together, the available preliminary literature suggests that SRC is associated with patient-specific impairments in CVR that are detectable by transcranial Doppler and MRI-based techniques during acute injury but, to date, only by MRI-based techniques during the subacute and chronic phases of injury. Results from MRI-based studies suggest that SRC may be characterized by a predominant pattern of increased CVR among acute and subacute patients as well as those that develop autonomic/physiological PCD and a predominate pattern of decreased CVR among patients that develop more chronic symptoms. Interestingly, the results of the available MRI-based CVR studies do not provide supportive evidence of a correlation between the severity of postconcussion symptoms and the magnitude of CVR alterations detected among SRC patients at different stages of recovery. Most importantly, this study confirms that brain MRI CO_2_ stress testing is well tolerated in patients with acute and subacute SRC and may be capable of quantifying the extent of CVR impairments in individual SRC patients.

The results of the present study must be considered in light of several important limitations. First, the sample size is relatively small and included adolescent patients who were recruited from a multidisciplinary pediatric concussion program. Patients evaluated at tertiary concussion programs are likely to have sustained more severe injuries that take longer to recover and therefore may be more likely to exhibit a greater impairment in CVR compared to a more generalized population of adolescent SRC patients. Second, the patient population examined in this study included those with a significant history of previous concussions. Although approximately 50% of patients that present to our multidisciplinary pediatric concussion program have a history of previous concussion (33), the lifetime burden of concussions among the SRC patients evaluated in this study may have had an impact on the magnitude of CVR alterations detected in this cohort. Indeed, preliminary longitudinal studies suggest that the cerebrovascular disturbances that accompany SRC may persist beyond symptomatic recovery (22, 34, 35). Third, the SRC patients in this study were imaged at non-uniform time points following injury, thus limiting direct comparisons between individual subjects. As such, future prospective longitudinal studies incorporating larger samples of adolescents and adults without a history of previous concussion who are imaged at uniform time points are needed to evaluate the impact of individual concussions on CVR alterations during the acute and clinically recovered stages of injury. Fourth, the incorporation of individualized masks during imaging postprocessing removed from analysis some of the prefrontal cortex and periventricular white matter, thus potentially resulting in an under-detection of abnormal voxels within these regions. Fifth, one of the most important limitations of this neuroimaging assessment technique is the effect of subject motion on accurate CBF and CVR measurements. It is well documented that both BOLD EPI and arterial spin-labeling techniques are highly susceptible to head motion (36, 37), which can have a confounding influence on BOLD and pCASL MRI CVR measurements. Although ongoing modifications to our brain MRI CO_2_ stress testing technique, including a reduction in the magnitude of the CO_2_ stimulus, have helped reduce the work of breathing and head movement during imaging acquisition, this limitation did result in the exclusion of a number of participants from the present study. Future studies should consider the use of head restraints and preemptive motion correction techniques (38, 39) as well as ramp CO_2_ breathing sequences (40, 41) that may be more tolerable than the abrupt changes associated with square-wave sequences as used here. Progressive CO_2_ breathing sequences will also allow help facilitate the measurement of dynamic cerebrovascular responses to hypercapnic stimuli (42). Sixth, although the leave-one-out ROC analysis in this study suggests that quantitative increases in CVR may be a promising biomarker to reliably distinguish between SRC patients and normal control subjects, quantitative voxel cut points will likely vary across future studies as a consequence of institutional and study differences in SRC patients, normal control subjects, and the vasoactive stimuli and neuroimaging platforms utilized. These methodological issues should be carefully considered when comparing results across previous and future CVR studies in this patient population. Finally, the neuroimaging assessment techniques utilized in this study only allowed for accurate and reliable measurement of global mean resting CBF and CVR and did not assess for other mechanisms that may contribute to the pathophysiology of TBI and patient symptoms including alterations in white matter integrity and resting state functional brain networks (7, 8) which may have accounted for the small false-positive and false-negative rates observed in this study and may have contributed to the lack of correlation observed between CVR alterations and symptom severity scores in the SRC patients. This later finding is not surprising; however, given the heterogeneity of pathophysiological processes (i.e., coexisting vestibulo-ocular and cervical spine dysfunction) (6) and non-injury patient factors (somatization, resilience) (43–45) that have been found to contribute to symptom severity following concussion and mTBI. Consequently, future studies combining brain MRI CO_2_ stress testing with other advanced neuroimaging techniques such as quantitative susceptibility-weighted imaging, DTI, and resting state fMRI are needed to provide a more comprehensive assessment of the complex pathophysiological mechanisms mediating SRC and mTBI and examine the potential diagnostic and clinical utility of these multimodal neuroimaging tools in individual patient diagnosis and management.

In conclusion, this study provides evidence that acute and subacute SRC is associated with patient-specific alterations in CVR that occur in the presence of normal global mean resting CBF. This study suggests that with meticulous attention paid to end-tidal gas control and head motion, brain MRI CO_2_ stress testing is capable of detecting and quantifying abnormal CVR among individual SRC patients over a wide range of injury acuities and postinjury time points. The results from our preliminary leave-one-out ROC analysis suggests that alterations in CVR detected by brain MRI CO_2_ stress testing may be a useful qualitative and qualitative biomarker to help reliably identify individual subjects with SRC. Larger prospective studies are needed to evaluate the clinical utility of brain MRI CO_2_ stress testing in the diagnosis, prognosis, and management of individual concussion and mild TBI patients.

## Ethics Statement

Institutional research ethics approval was obtained for this study. Informed patient, and where applicable, parental consent was obtained for all participants prior to participating in the study. The approving body was the Biomedical Research Ethics Board of the University of Manitoba, Max Rady College of Medicine.

## Author Contributions

WM and ME: conception of study, conduct of study, analysis of data, interpretation of data, writing, and final approval. LR: conduct of study, analysis of data, interpretation of data, writing, and final approval. PM: conception of study, interpretation of data, writing, and final approval. MM and PP: conduct of study, writing, and final approval. ME: conduct of study, interpretation of data, writing, and final approval. DM, JD, and JF: interpretation of data, writing, and final approval.

## Conflict of Interest Statement

JF, JD, and DM are senior scientists at Thornhill Research Inc. (TRI), a company affiliated with the University Health Network that developed the RespirAct™, a non-commercial research tool assembled by TRI to enable cerebrovascular reactivity studies. The other authors have no financial disclosures or conflicts of interest to declare.

## Supplementary Material

Figure S1 The correlation between abnormal voxel counts and PCSS scores. Abbreviation: PCSS, Postconcussion Symptom Scale.

Figure S2 Histogram demonstrating the distribution of group mean CVR for the sport-related concussion patients and healthy control subjects. The absolute CVR (deltaBOLD/deltaCO_2_) was 0.223 ± 0.127 for the sports-related concussion patients and 0.139 ± 0.079 for the control subjects.
