# Supplementary figures and images for: Patient-Specific Alterations in CO2 Cerebrovascular Responsiveness in Acute and Sub-Acute Sports-Related Concussion
Source: Front Neurol. 2018 Jan 24;9:23. doi: 10.3389/fneur.2018.00023 (PMC5787575; doi:10.3389/fneur.2018.00023)

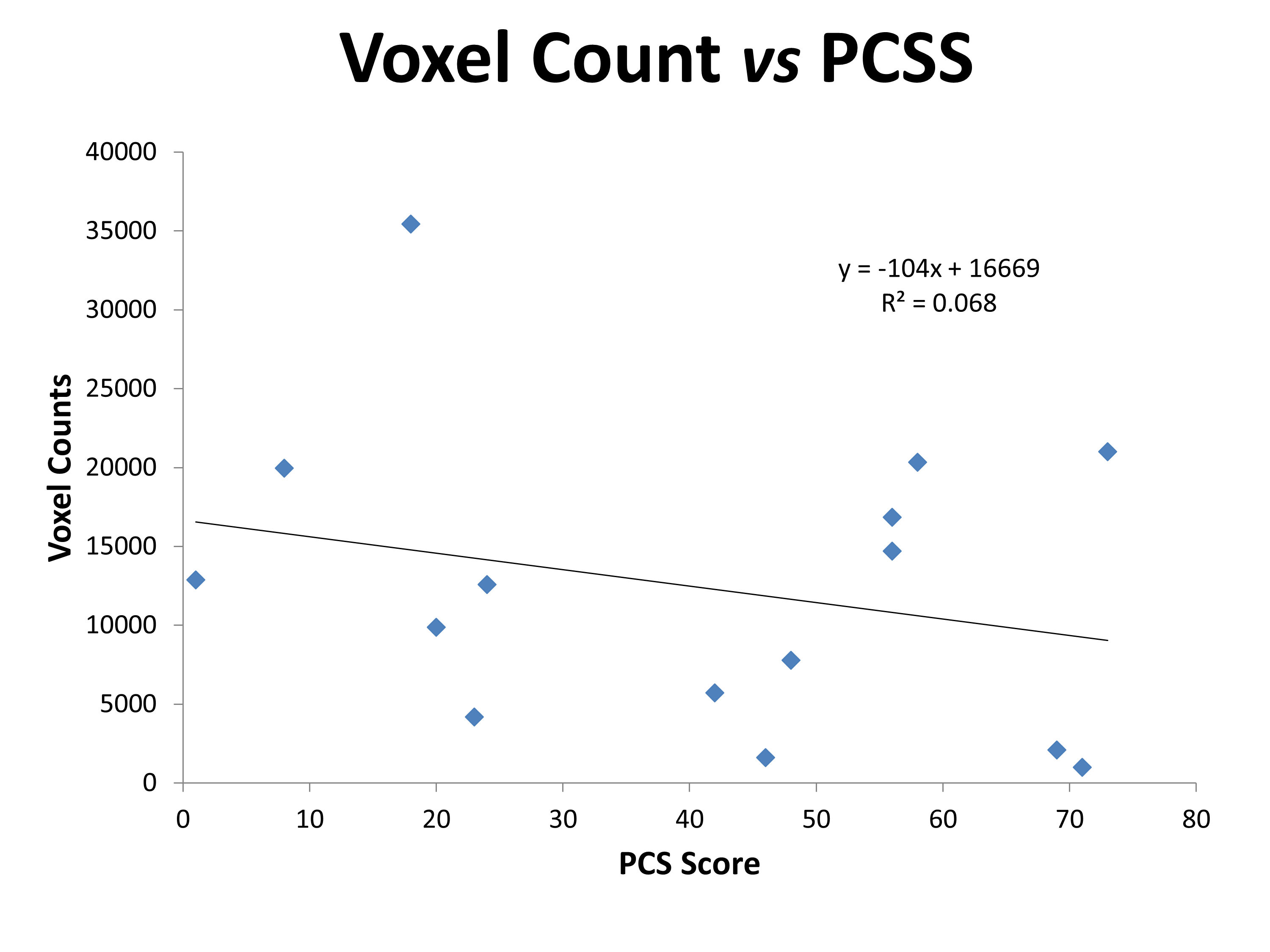

Supplement: Figure S1 — The correlation between abnormal voxel counts and PCSS scores. Abbreviation: PCSS, Postconcussion Symptom Scale. [file image_1.tif]

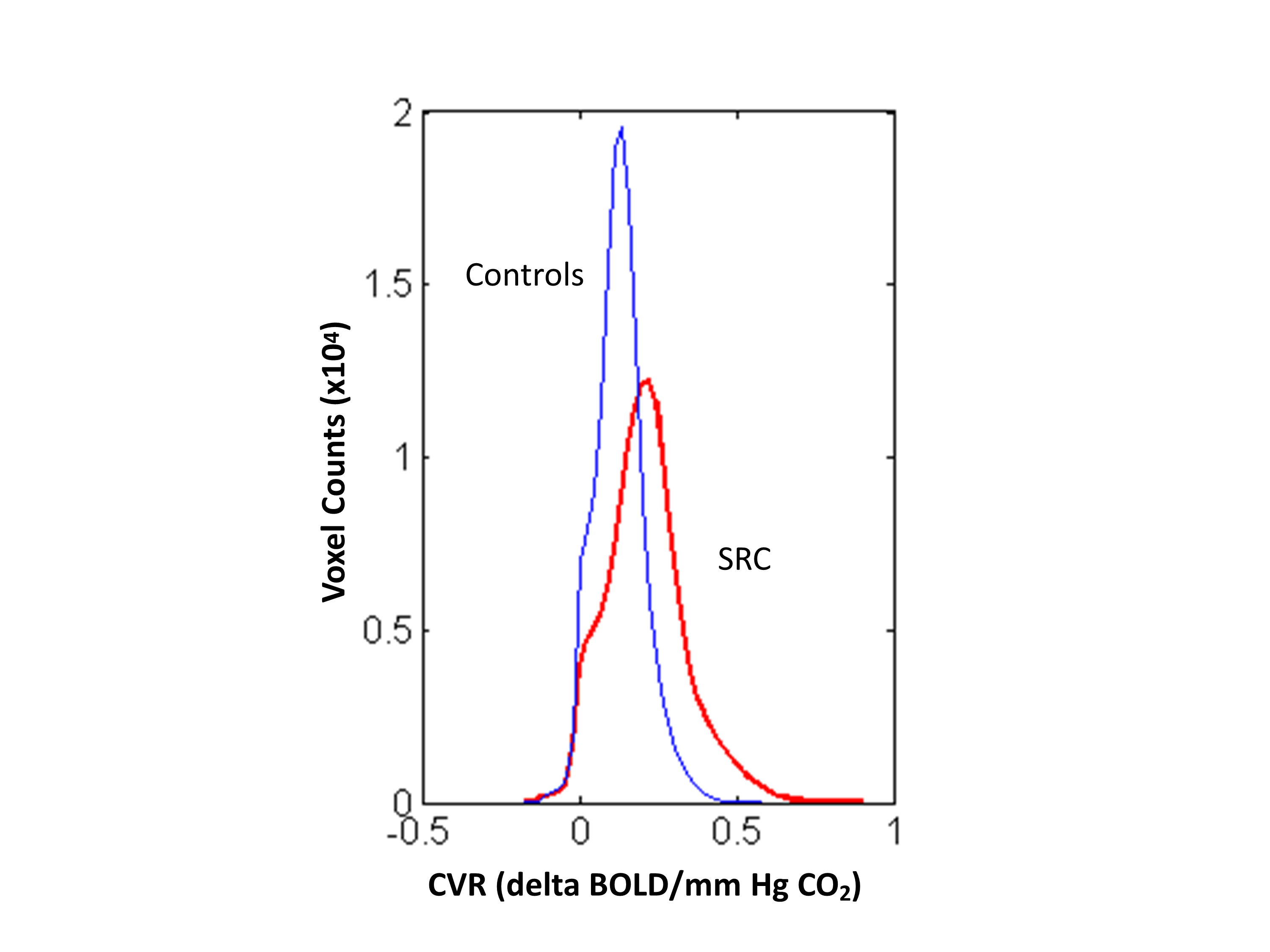

Supplement: Figure S2 — Histogram demonstrating the distribution of group mean CVR for the sport-related concussion patients and healthy control subjects. The absolute CVR (deltaBOLD/deltaCO2) was 0.223 ± 0.127 for the sports-related concussion patients and 0.139 ± 0.079 for the control subjects. [file image_2.tif]
